# Supplementary material for: MetaRibo-Seq measures translation in microbiomes
Source: Nat Commun. 2020 Jun 29;11:3268. doi: 10.1038/s41467-020-17081-z (PMC7324362; doi:10.1038/s41467-020-17081-z)
Supplement: Supplementary file 10 — Supplementary Data 7 [file 41467_2020_17081_MOESM10_ESM.zip › File2/Confidence_High_Taxonomy/320418_out.krona.html]

Javascript must be enabled to view this page.

members
magnitude
magnitudeUnassigned
count
unassigned
taxon
rank

320418\_out

72

2
72
superkingdom

phylum
976
72

class
200643
72

171549
72
order

1
171552
family

genus
838
1

species
1

SRS018888\_contig\_number\_7599
165179

family
2005525
61

genus
1
375288

SRS893172\_contig\_number\_966
61

species
58
46503

SRS013951\_contig\_number\_31865SRS015264\_contig\_number\_16984SRS015694\_contig\_number\_15249SRS016056\_contig\_number\_747SRS016132\_contig\_number\_1930SRS016753\_contig\_number\_3323SRS017433\_contig\_number\_11666SRS017701\_contig\_number\_9744SRS018133\_contig\_number\_12896SRS019030\_contig\_number\_17938SRS019161\_contig\_number\_19536SRS019286\_contig\_number\_2718SRS020233\_contig\_number\_28912SRS023526\_contig\_number\_12193SRS023583\_contig\_number\_5014SRS042628\_contig\_number\_37051SRS043667\_contig\_number\_1228SRS045739\_contig\_number\_4077SRS047433\_contig\_number\_18703SRS048164\_contig\_number\_22828SRS048870\_contig\_number\_11248SRS049712\_contig\_number\_18781SRS050026\_contig\_number\_193SRS050299\_contig\_number\_9474SRS050520\_contig\_number\_44SRS050941\_contig\_number\_18486SRS051882\_contig\_number\_19691SRS054905\_contig\_number\_18798SRS055966\_contig\_number\_338SRS058770\_contig\_number\_4462SRS062654\_contig\_number\_27544SRS063127\_contig\_number\_639SRS074670\_contig\_number\_2919SRS075773\_contig\_number\_14609SRS076976\_contig\_number\_16997SRS077024\_contig\_number\_4565SRS077086\_contig\_number\_8177SRS077552\_contig\_number\_15556SRS077641\_contig\_number\_16455SRS077849\_contig\_number\_15397SRS078419\_contig\_number\_12732SRS097889\_contig\_number\_37280SRS098644\_contig\_number\_39607SRS1041031\_contig\_number\_12625SRS1041130\_contig\_number\_3914SRS1041140\_contig\_number\_20856SRS1055022\_contig\_number\_1798SRS143780\_contig\_number\_27208SRS143876\_contig\_number\_13222SRS143991\_contig\_number\_24053SRS144135\_contig\_number\_25840SRS146764\_contig\_number\_29187SRS146812\_contig\_number\_59428SRS146813\_contig\_number\_23928SRS147139\_contig\_number\_17113SRS149181\_contig\_number\_26759SRS893230\_contig\_number\_6319SRS893327\_contig\_number\_5244

387661

SRS015217\_contig\_number\_7163SRS016018\_contig\_number\_8754
2
species

1
1853231
family

genus
283168
1

28118

SRS012969\_contig\_number\_15705
1
species

171550
1
family

239759
1
genus


SRS075821\_contig\_number\_contig-100\_15.125310
1118061
1
species

family
8
815

8
816
genus

1

SRS049959\_contig\_number\_49077
291644
species

species
1
28116

SRS078665\_contig\_number\_9344

1638779

SRS098571\_contig\_number\_21384
1
species

species
46506

SRS065397\_contig\_number\_5814
1

310297

SRS105082\_contig\_number\_688
1
species

species
47678

SRS145497\_contig\_number\_10192
1

1

SRS044535\_contig\_number\_409
1946088
species

1
817

SRS104636\_contig\_number\_26322
species
